# Cancer-related effects on relationships, long-term psychological status and relationship satisfaction in couples whose child was treated for leukemia: A PETALE study

**DOI:** 10.1371/journal.pone.0203435

**Published:** 2018-09-07

**Authors:** Willow Burns, Katherine Péloquin, Émélie Rondeau, Simon Drouin, Laurence Bertout, Ariane Lacoste-Julien, Maja Krajinovic, Caroline Laverdière, Daniel Sinnett, Serge Sultan

**Affiliations:** 1 Department of Psychology, Université de Montréal, Montréal, Québec, Canada; 2 Sainte-Justine University Health Center, Montréal, Québec, Canada; 3 Department of Pediatrics, Université de Montreal, Montréal, Québec, Canada; University Medical Center Groningen, University of Groningen, NETHERLANDS

## Abstract

**Objectives:**

Follow-up studies suggest that the psychosocial impact of pediatric cancer on parents often extends beyond the end of their child’s cancer treatments, and parents can continue to experience both individual and relationship effects. In a long-term study of parents of children who were treated for acute lymphoblastic leukemia (ALL), we aimed to: 1) describe parents’ adjustment (psychological distress, relationship satisfaction; 2) describe the perceived impact of cancer on couples’ relationship, and; 3) identify to what extent the perceived impact of cancer on the couple is related to both parents’ long-term adjustment.

**Methods:**

Parents of childhood ALL survivors (*n* = 103 couples) were surveyed as part of a cohort recall (PETALE cohort). Both parents completed questionnaires exploring adjustment (Brief Symptom Inventory-18, Dyadic Adjustment Scale) and perceived impact of cancer on the relationship (Impact of Cancer on the Couple). Mothers’ and fathers’ scores were compared using MANOVAs. We also examined the degree to which a parent’s perceived changes in relationship dynamics following their child’s cancer were associated with their own current adjustment (actor effects), and their partner’s current adjustment (partner effects) using the Actor-Partner Interdependence Model (APIM).

**Results:**

Frequencies of current distress were normative in parents (mothers/fathers): general distress (6.8/7.8%), anxiety (5.8/6.8%), depression (2.9/6.8%), somatization (13.6/9.7%), and relationship distress (21.4/20.4%). Mothers and fathers typically agreed on their reported relationship satisfaction, and the perceived nature of relationship changes following the illness. Dyadic analyses indicated that whereas mothers’ adjustment was related to their own perceived relationship changes, fathers’ adjustment was primarily related to their partner’s perceptions.

**Conclusion:**

In long-term stable couples, mothers may act as an influential bridge connecting the illness experiences of survivors and fathers. This could explain why mothers’ perceptions of relationship changes were related to their partners’ long-term adjustment, which was not the case for fathers.

## Introduction

Childhood cancer has been identified as a long-term vulnerability factor for parents’ well-being at both the individual level [1–5] and the level of parents as a couple [6–9]. Although reports have documented individual distress levels in parents, few have compared both parents in the couple and explored dyadic interrelations within couples. In addition, no studies have systematically surveyed the perceived impact of cancer on parents’ relationship and how this may explain the current adjustment of parents several years after the illness has subsided. In this study, we aimed to assess long-term psychological status and relationship satisfaction in parents of childhood leukemia survivors, explore their perceived impact of cancer on their relationship, and how this impact may explain both parents’ current individual and relationship adjustment. A recent review on parents of childhood cancer survivors suggested that although most parents reported normal ranges of psychological distress, a significant subgroup reported clinically significant distress [4], with 21–44% of parents reporting severe posttraumatic stress symptoms (PTSS). In contrast, within a recent cross-sectional study of parents of long-term acute lymphoblastic leukemia (ALL) survivors, clinically significant anxiety, depression, and posttraumatic stress were reported by 7.1%, 3.1%, and 3.9% of parents, respectively [5]. Thus, there appears to be great variability in the proportion of parents reporting significant distress as indicated by different studies. Consequently, this might suggest that select factors may explain these varying rates of distress. Factors associated with heightened distress in parents have been identified. First, the time elapsed since the child’s diagnosis has been reported as a factor associated with parental distress, such that distress typically decreases as more time passes [4, 10]. Second, parents’ use of maladaptive coping strategies earlier in the illness and their child’s poor adjustment, have also been found to predict parents’ long-term or late effects [4]. Third, a constructive social context surrounding the illness, such as better family functioning and availability of social support, can also help attenuate parents’ distress [4, 10]. Indeed, parents in conflictive families tend to report more anxiety, depression, and PTSS, while cohesive families tend to report less depression symptoms [11]. Additionally, parents’ gender was a significant factor, with mothers reporting more distress especially early in the illness trajectory [4, 10].

Another factor, which may explain the current psychological status of parents, is the impact of cancer on their relationship. A few select reports have investigated the impact of cancer on the relationship of the parental couple. Some couples emphasize that the illness had a positive impact on their relationship (e.g., greater trust, communication, support, and emotional closeness), whereas others emphasize its negative impact (e.g., deteriorations in sexuality) [6–8, 12]. To date only two empirical studies have directly assessed which aspects of the parents’ relationship changed over the illness trajectory and the extent to which the cancer experience challenged or tested their relationship [8, 12]. However, these studies found contrasting results. One multicenter cross-sectional survey of parents of children currently being treated for cancer (who were at least 3 months post-diagnosis) or in follow-up care (completed cancer treatment within past 3 years) (*N* = 192 parents; 122 mothers and 70 fathers) found that a third of parents experienced deterioration in their relationship quality and low dyadic adjustment, with more than half claiming that their relationship as a couple had been challenged following the illness [8]. A smaller cross-sectional study (*N* = 35 couples) found that spouses whose child with cancer was between 1 to 7 years post-diagnosis experienced strengthened communication, deteriorations in sexuality, and no change on relationship dimensions of conflict resolution, leisure activities, and division of household labour [12]. A recent review on couples’ functioning following their child’s cancer diagnosis parallels these mixed findings on conflict reported by both qualitative and quantitative studies [7]. Notably, no reports have explored the impact of cancer on important relationship areas such as partner support and intimacy. As exemplified in these reports, parents’ adjustment to cancer has most often been studied with each partner analyzed separately. For instance, a longitudinal study of parents of children with cancer found that for both mothers and fathers their marital adjustment at follow-up was partially explained by their spouse’s marital satisfaction scores. Although this study can boast that it considered the association between one spouse’s marital satisfaction and the other spouse’s marital adjustment, this study was not truly dyadic in its design. Hierarchical multiple regression analyses were conducted separately for mothers and fathers. Interdependence in the couples’ data and potential gender differences in these associations were also not statistically accounted for or tested [13]. Nevertheless, this study was conducted at 2 and 20 months post-diagnosis; hence it does provide a basis for assuming that relationship dynamics during the child’s treatment could also be associated with parents’ relationship adjustment in the survivorship period.

Recent studies have started to address dyadic interrelationships in parents of children with cancer. However, only five empirical studies in the field of pediatric cancer have been conducted thus far [9, 14–17], and of these only two had longitudinal designs [9, 17]. Notably, the first longitudinal study found that mothers’ relationship adjustment two years after their child’s ALL diagnosis was associated with their own perception of family support, role conflict, and role overload at diagnosis. Fathers’ relationship adjustment was associated with both their own perceptions (role conflict, role ambiguity, being tired) and their partner’s perception (role conflict) of family functioning at diagnosis [9]. The second study examined posttraumatic stress symptoms (PTSS) among couples from diagnosis to one year after the end of the child’s treatment. Findings from this study suggested interdependence in partners’ PTSS during the child’s cancer treatment, but not after the end of treatment [17]. To date, no longitudinal or dyadic assessment has been used to articulate the perceived relationship impact of cancer on partners’ current emotional adjustment or relationship quality.

Our research objectives were threefold. We aimed to: 1) Describe the psychological and relationship adjustment (psychological distress, relationship satisfaction) of mothers and fathers whose child was treated for acute lymphoblastic leukemia. To complement previous reports, we wished to study parents’ adjustment long after children’s remission (>5 years post-diagnosis). 2) Describe the perceived impact of cancer on these couples using a systematic approach of couples’ functioning including core relationship dimensions such as intimacy, partner support, sexuality, conflict, or shared time and activities. 3) Identify to what extent the perceived impact of cancer on the couple is related to both parents’ long-term adjustment (psychological distress, relationship satisfaction). To bridge the gap in the existent literature, we examined actor (i.e., the effect of one’s perceived impact of cancer on their own adjustment) and partner (i.e., the effect of a parent’s perceived impact of cancer on the other parent’s adjustment) effects, as well as gender differences in these effects.

## Materials and methods

### Participants

Participants were parents of childhood ALL survivors from the PETALE cohort [18]. Childhood ALL survivors that were diagnosed and treated at Sainte-Justine University Health Centre (SJUHC, Montreal, Canada), with DFCI protocols 87–01 to 2005–01, and their parents were recruited to participate in this long-term follow-up study (224 families). In order to be eligible to participate in the PETALE study, ALL survivors had to: (a) be less than 19 years of age at the time of their diagnosis; (b) not having received a transplant and not having experienced relapse or a second cancer, and; (c) be at least 5-years post-diagnosis at the time of recall.

Given the objectives of this study, families were excluded if the data was only available for the survivor but not their parents (*n* = 31) or for only one parent (*n* = 84). Parents who were not caring for their child during the illness or couples that were separated either during their child’s cancer treatments or at the time of cohort recall were also excluded (*n* = 6). The final sample was thus comprised of 103 ‘intact couples’ (i.e., stable couples that were together both during their child illness and at the time of this cohort recall; see **S1 Fig**), resulting in a final participation rate of 46% (103 / 224 families).

On average, mothers and fathers were 51 and 54 years old respectively and survivors were 22 years old at the time of recall. Survivors were also on average 15 years post-diagnosis and slightly more than half reported known late-adverse effects at the time of assessment (**Table 1**).

**Table 1:** Parents’ and childhood ALL survivors’ characteristics (*n* = 103).

**A**		
**Parents’ characteristics**	**Mothers** **M (SD) or N (%)**	**Fathers** **M (SD) or N (%)**
Length of relationship, years	29.90 (7.63)	29.90 (7.63)
Age at diagnosis, years	35.76 (5.83)	37.70 (5.12)
Age at follow-up interview, years	51.56 (6.75)	53.63 (6.11)
Highest education level		
High school	21 (20.4)	37 (35.9)
Undergraduate	50 (48.5)	46 (44.7)
Graduate	12 (11.7)	7 (6.8)
Other (e.g., high school not completed)	20 (19.4)	13 (12.6)
Primary occupation		
Working, full-time	65 (63.1)	79 (76.7)
Working, part-time	12 (11.7)	4 (3.9)
Other (e.g., retired, unemployed, at home)	26 (25.2)	20 (19.4)
Financial income (gross, $CAD)		
< $49,999	67 (65.0)	30 (29.1)
$50,000–89,999	30 (29.1)	42 (40.8)
$90,000 +	6 (5.8)	31 (30.1)
Language		
French	98 (95.1)	97 (94.2)
English	0 (0)	1 (1.0)
Other	5 (4.9)	5 (4.9)
**B**		
**Survivors’ characteristics**		
Age at diagnosis, years	6.26 (4.78)	
Age at follow-up interview, years	22.09 (6.66)	
Child (≤ 18)	41 (39.8)	
Adolescent/young adult (19–25)	35 (34.0)	
Adult (≥ 26)	27 (26.2)	
Time since diagnosis, years	15.46 (5.12)	
Range	5–27	
Time since end of treatment, years	13.28 (5.20)	
Range	3–25	
Sex		
Female	59 (57.3)	
Male	44 (42.7)	
ALL relapse risk group		
Standard risk	45 (44.1)	
High risk	57 (55.9)	
Treatment protocol		
DFCI 87–01	10 (9.7)	
DFCI 91–01	19 (18.4)	
DFCI 95–01	34 (33.0)	
DFCI 2000–01	32 (31.1)	
DFCI 2005–01	8 (7.8)	
Radiotherapy		
No	39 (37.9)	
Yes	64 (62.1)	
Known long-term complications		
No	45 (44.1)	
Yes	57 (55.9)	
Relationship status		
Single	73 (70.9)	
Married	4 (3.9)	
Divorced	2 (1.9)	
Common law partner	24 (23.3)	
Highest education level		
High school not yet completed	34 (33.0)	
High school	19 (18.4)	
Undergraduate	37 (35.9)	
Graduate	1 (1.0)	
Other (e.g., vocational diploma)	12 (11.7)	
Financial income (gross, $CAD)		
< $49,999	87 (84.5)	
$50,000–89,999	15 (14.6)	
$90,000 +	1 (1.0)	
Primary occupation		
Working, full-time	39 (37.9)	
Working, part-time	30 (29.1)	
Other (e.g., student, unpaid work, unemployed)	34 (33.0)	
Language		
French	98 (95.1)	
English	2 (1.9)	
Other	3 (2.9)	

### Procedures

The research coordinator or clinical research assistant invited eligible families from the PETALE cohort to participate in this recall study by phone. The ALL survivors who agreed to participate came to the hospital for a day of testing. Parents completed a series of questionnaire on site if they accompanied their child to the hospital, or at home and returned them by mail if they were not present. Both parents were invited to participate and were asked to complete their questionnaires independently. The research coordinator called the parents for a follow-up if the questionnaires were not returned within 3 weeks. Data were checked for clinically significant distress and appropriate referrals were made when deemed necessary in order to comply with ethical standards. The research coordinator or clinical research assistant would call parents to collect missing data. Survivors' medical information was collected from patients’ medical records. The research coordinator collected survivors’ socio-demographic information during the day of testing. Data presented in this report were collected from February 2013 to May 2016. All participants provided informed consent and the Institutional Review Ethics Board at SJUHC approved the study. Further description of this cohort is available in a previous report [18].

### Measures

#### Brief Symptom Inventory-18 (BSI-18) [19]

The Brief Symptom Inventory is an 18-item self-report questionnaire, assessing psychological distress [19]. Previous studies have also specifically used this measure with cohorts of adolescent and adult survivors of childhood cancer [20, 21], as well as with their parents [22]. It includes three symptom dimensions: Anxiety, Depression, and Somatization, as well as a total score, the Global Severity Index (GSI), which reflects an individual’s global level of distress. Respondents are asked to report on their symptoms in the past 7 days. They can be classified with their standardized T-scores as either being at a high risk for psychological distress symptoms (i.e., *positive caseness*; T_GSI_ ≥ T_63_ or T_2DIMENSIONS_ ≥ T_63_) or not being at any apparent risk (i.e., *negatives caseness*). Norms are based on adult community samples and are available across gender and ages [19]. Internal consistency in the current sample was adequate (*α*_anxiety_ = .86; *α*_depression_ = .88; *α*_somatization_ = .74; *α*_global severity_ = .93).

#### Dyadic Adjustment Scale (DAS-4) [23]

The abbreviated Dyadic Adjustment Scale (DAS-4) [23] evaluates current relationship satisfaction using four items Although this exact version of the Dyadic Adjustment Scale (DAS-4) has been widely cited in the field of couples’ research [24–26], it has not previously been used with parents of children with cancer or parents of childhood cancer survivors. However, studies on couples that have a partner with cancer have used the original 32-item Dyadic Adjustment Scale (DAS) [27–29] or brief versions [30–33]. To date, only one empirical study has used a form of the Dyadic Adjustment Scale (14 items) with parents of children with cancer that are either actively being treated or have completed cancer treatments [8]. A global DAS-4 score is calculated by summing the four items (range 0–21), with a higher score thereby suggesting greater relationship satisfaction. Using community samples of married and cohabiting couples and couples seeking relationship therapy, the DAS-4 has been found to effectively classify couples as clinically distressed (DAS < 13) or non-distressed (DAS ≥ 13), as well as predict couples’ dissolution over time [23]. The internal consistency was adequate in the current study (*α =* .84).

#### Impact of cancer on the couple [34]

The Impact of Cancer on the Couple is a brief survey composed of 7 items that was developed specifically in the context of the current study to assess the perception of changes in several relationship dimensions following a child’s cancer diagnosis [34]. The first few items of the questionnaire were simply used for contextualization and screening purposes in this study (e.g., screening out parents who were separated, divorced, widowed or in a relationship with another partner who was not the parent of the childhood ALL survivor). Parents were asked to reflect back on the time that their child was in treatment and to rate the impact of their child’s illness on six dimensions of their relationship with their partner: Intimacy, Quality of partner support, Sexuality, Conflict, Time spent together and activities, and Relationship satisfaction. Each relationship dimension is rated on a continuum ranging from “*1 = very negative effect*” to “*7 = very positive effect*”. A score of 4 reflects “*no change”*. When parents report a negative effect (scores 1 to 3), they are asked to indicate the extent to which these negative effects persisted once cancer treatment ended (“*The negative effects disappeared immediately”; “The negative effects remained but faded over time”; “The negative effects were permanent”*). To describe the nature of the changes experienced by parents, dimension scores were also classified into three main categories: negative change (scores of 1 to 3), no change (score of 4), and positive change (scores of 5 to 7). Parents were also asked to assess the Overall perceived impact of their child’s illness on their relationship, and quantify this change on a 1 to 7 scale, ranging from *“1 = this period has distanced us/has been detrimental to our relationship”* to *“7 = this period brought us closer/strengthened our relationship*.*”* A score of 4 on this particular item signifies *“this period had no effect on our relationship*.*”* For the purposes of consistency and ease of visual representation, the same classifications as above were used to denote negative change, no change and positive change on this item. The scale showed good internal consistency in the current study (*α* = .84). The original French questionnaire (**S1 File**) and a translated English version (**S2 File**) are available for download as supplementary files to this article.

### Statistical analyses

The distributions of all variables were assessed for normality. For Objectives 1 and 2 no transformations were applied. For Objective 3, non-normally distributed variables (skewness and kurtosis > 1) were subjected to the following non-linear transformations: a reflection and square root transformation on Quality of partner support (Impact of Illness on the Couple), a square root transformation on Global distress (Global Symptom Index—BSI-18), and inverse transformations on Anxiety, Depression, and Somatization (BSI-18). Depression variables were severely skewed, and parents in three couples had extreme depression scores (*Z* score > 3.5). Data from these three couples were retained in all analyses for Objectives 1 and 2, because the emphasis was primarily descriptive. However, due to normality concerns they were excluded from the dyadic analyses in Objective 3. In the remaining couples, even after inverse transformations on both partners’ depression variables were applied the resulting distributions were still slightly skewed but since these distributions more closely approached normality, these inverse transformations were used in the dyadic analyses. The remaining variables were all normally distributed. There was no missing data. To detect possible control variables among clinical and demographic variables for Objective 3 (age of child at diagnosis, age of parents at diagnosis, age of survivor at follow-up, age of parents at follow-up, relationship length, time elapsed since diagnosis, time elapsed since end of treatment, sex of child, ALL risk group, use of radiotherapy, long-term complications), we conducted bivariate correlations and repeated-measures MANOVAs (where gender served as a repeated-measure for the couple). Given that no significant associations were found no covariates were included in the main analyses.

#### Objective 1: Description of parents’ long-term adjustment

In order to compare mothers and fathers on adjustment variables, a repeated-measures MANOVA was conducted, where gender served as a repeated-measure for the couple. To assess the degree to which mothers and fathers resemble each other on adjustment variables, intra-class correlation coefficients (ICCs) were also calculated. ICC values were categorized as either: poor (ICC < .40), fair (ICC = .40-.59), good (ICC = .60-.74), or excellent (ICC = .75–1.00) [35]. Next, we calculated the proportion of mothers and fathers reporting clinically significant scores on each adjustment variables. McNemar tests were used to compare the proportion of mothers and fathers meeting criteria for positive caseness.

#### Objective 2: Description of perceived impact of cancer on the couple

**Fig 1:**
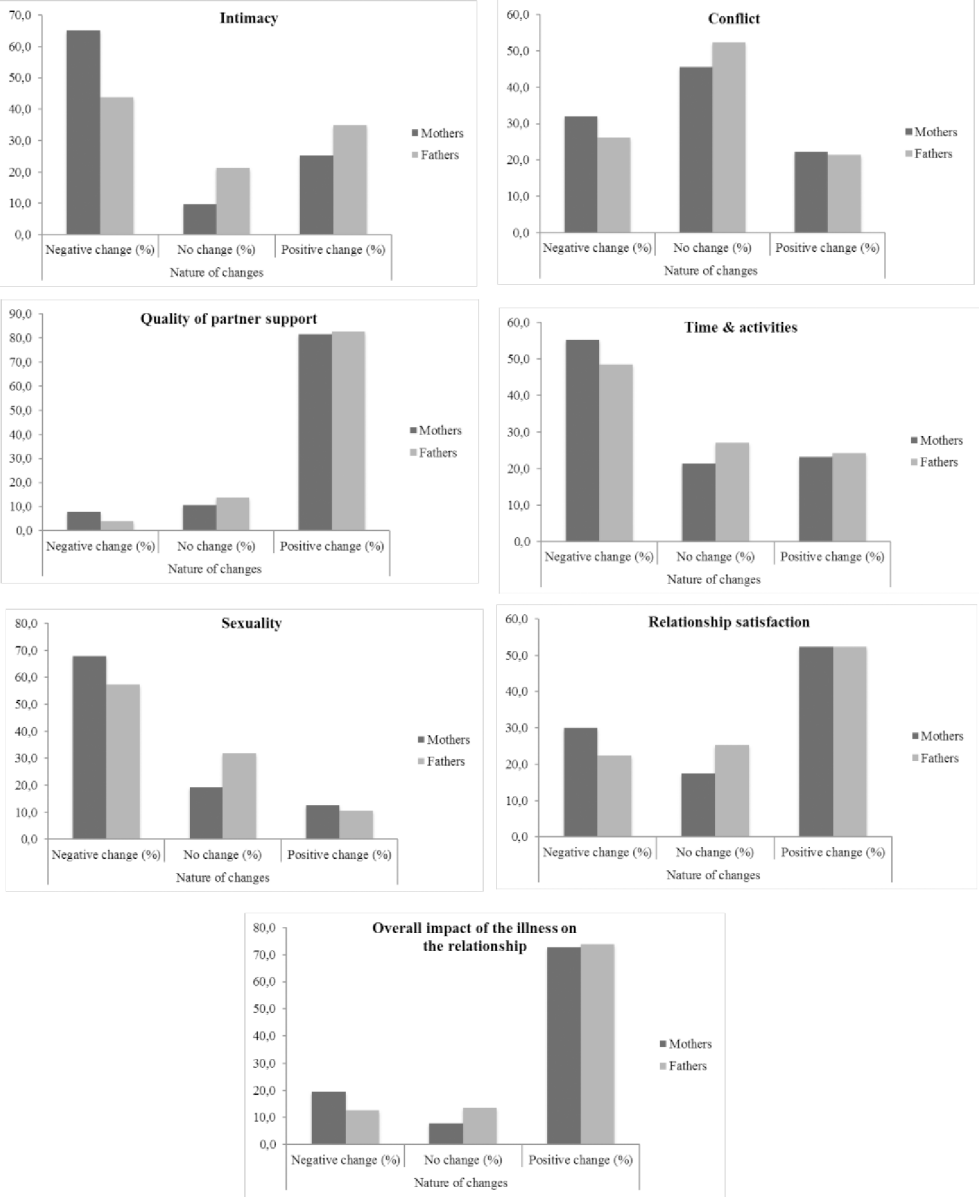
Bar charts displaying the nature of relationship changes for mothers and fathers (*n* = 103). *Note*. Relationship dimensions are represented on a 1 to 7 Likert scale, whereby participants’ scores are classified into: negative change (scores of 1–3), no change (scores of 4), or positive change (scores of 5–7).

The same strategy was applied to compare mothers’ and fathers’ perceived changes in their relationship. Wilcoxon tests and bar chart comparisons (see **Fig 1**) were used to compare the proportion of mothers and fathers that reported each type of relationship dimension change (negative change, no change, positive change).

#### Objective 3: Dyadic models for long-term adjustment

**Fig 2:**
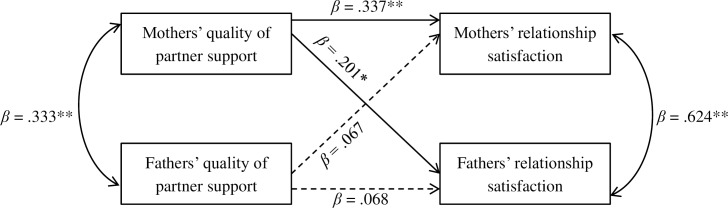
Actor-partner interdependence model (APIM) predicting current relationship satisfaction from perceived changes in quality of partner support (*n* = 100 couples). *Note*.** p < .01, * p < .05; the dashed lines represent non-significant associations.

Dyadic associations among cancer-related relationship changes, psychological distress, and relationship satisfaction were examined using the Actor-Partner Interdependence Model (APIM) with SPSS MIXED MODELS, a modified regression-based technique which allows for prediction of outcome variables among dyads (see **Fig 2**) [36]. This multilevel modeling approach has several advantages over traditional regression analyses: a) accounting for the non-independence of couples’ data; b) simultaneously testing both actor and partner effects, and; c) testing gender differences in the strength of actor and partner effects [36]. Analyses were conducted to predict parents’ current relationship satisfaction and psychological distress (Global Severity Index, Anxiety, Depression, and Somatization symptoms) from both partners’ perception of relationship changes following their child’s cancer treatments (Impact of Cancer on the Couple). We conducted separate models for each predictor (dimensions of relationship changes), and each outcome variable (see **Fig 2** for an example of one such APIM model). To explore potential gender differences, gender and the interaction between gender and predictors were included in all analyses. A significant interaction term indicates a significant gender difference in the strength of an actor or partner effect. Significance levels of *p* < .05 were set for all dyadic analyses.

## Results

### Objective 1: Description of parents’ long-term adjustment

Results of the overall MANOVA showed that mothers and fathers significantly differed on their level of relationship satisfaction and psychological distress (*F*(5, 98) = 5.70, *p* < .001, n_*p*_^2^ = .23), with fathers reporting greater depression symptoms than mothers. No gender differences were found for relationship satisfaction, global distress, and symptoms of anxiety and somatization (**Table 2**). Poor agreement between parents was found on global distress and all psychological distress symptoms (i.e., anxiety, depression, and somatization). In contrast, mothers and fathers exhibited excellent agreement with respect to their reports of relationship satisfaction. We found that 21.4% of mothers and 20.4% of fathers reported clinically significant relationship distress (non-significant difference) (**S1 Table**). A minority of parents scored within the clinical range on global distress (6.8% of mothers and 7.8% of fathers), anxiety (5.8% of mothers and 6.8% of fathers), depression (2.9% of mothers and 6.8% of fathers), and somatization (13.6% of mothers and 9.7% of fathers) (**S1 Table**). Differences between mothers’ and fathers’ frequencies were not significant (**S1 Table**).

**Table 2:** Description of the perceived impact of cancer, and psychological and relationship adjustment in a sample of 103 couples of parents whose children were treated for ALL (*n* = 103).

	Mothers *M (SD)*	Fathers *M (SD)*	Repeated-measures MANOVA testing gender differences	ICC (95% CI)	Levels of agreement
**Impact of Cancer on the Couple**					
Intimacy	3.55 (1.74)	3.94 (1.76)	*F*(1,102) = 3.768, *p* = .055	.487, *p* = .000 (.247-.651)	Fair
Quality of partner support	5.80 (1.43)	5.81 (1.19)	*F*(1,102) = .086, *p* = .770	.480, *p* = .001 (.230-.649)	Fair
Sexuality	3.17 (1.46)	3.20 (1.38)	*F*(1,102) = .079, *p* = .779	.683, *p* = .000 (.531-.785)	Good
Conflict	4.02 (1.16)	4.02 (1.11)	*F*(1,102) = .000, *p* = 1.000	.539, *p* = .000 (.317-.688)	Fair
Time & activities	3.55 (1.56)	3.66 (1.45)	*F*(1,102) = .322, *p* = .571	.329, *p* = .023 (.007-.547)	Poor
Relationship satisfaction	4.69 (1.75)	4.69 (1.46)	*F*(1,102) = .000, *p* = 1.000	.636, *p* = .000 (.462-.754)	Good
Overall impact on couple	5.49 (1.80)	5.58 (1.62)	*F*(1,102) = .360, *p* = .550	.703, *p* = .000 (.561-.799)	Good
**Dyadic Adjustment Scale (DAS-4)**					
Relationship satisfaction	15.36 (3.69)	15.55 (3.50)	*F*(1,102) = .441, *p* = .508	.796, *p* = .000 (.699-.862)	Excellent
**Brief Symptom Inventory (BSI-18)**					
Global Symptom Index (GSI)	46.11 (9.41)	46.39 (9.67)	*F*(1,102) = .058, *p* = .810	.251, *p* = .075 (-.111-.494)	Poor
Anxiety	47.38 (8.91)	45.56 (9.26)	*F*(1,102) = 2.839, *p* = .095	.070, *p* = .354 (-.362-.367)	Poor
Depression	**44.75 (7.33)**	**46.25 (7.66)**	*F*(1,102) = 3.986, *p* = .049	.212, *p* = .110 (-.151-.462)	Poor
Somatization	49.24 (8.39)	48.90 (8.43)	*F*(1,102) = .100, *p* = .753	.376, *p* = .009 (.076-.578)	Poor

*Note*. Means and standard deviations are computed using t-scores on the BSI-18. Bolded means indicate a significant gender difference. To facilitate interpretation, untransformed means and standard deviations are presented here

### Objective 2: Description of perceived impact of cancer on the couple

We found that mothers and fathers did not differ on their perception of relationship changes following cancer (*F*(7, 96) = .96, *p* = .47) (**Table 2**). Inspection of the ICC revealed that the levels of agreement between parents on the perceived impact of the illness ranged from poor to good agreement. Poor agreement was found on time spent together and activities, whereas fair agreement levels were found on intimacy, quality of partner support and conflict. Mothers and fathers reported good agreement on sexuality, relationship satisfaction, and the overall perceived impact of the illness on the couple (**Table 2**). The vast majority of parents (>75% of mothers and fathers) reported a positive impact on the quality of support with their partner, and more than 50% reported a positive impact on their relationship satisfaction (**S2 Table**). Roughly half of the parents reported that the illness had no significant impact on relationship conflict, whereas some parents reported that their child’s illness had a predominately negative effect on their relationship. For instance, a significantly greater proportion of mothers (65%) compared to fathers (43.7%) reported a negative impact of their child’s cancer on their level of intimacy with their partner (Wilcoxon z = - 2.861, *p* = .004) (**S2 Table**). These negative changes on intimacy disappeared immediately when their child’s treatments were completed or faded with time for most mothers (94%) and fathers (97.7%). A similar trend was observed for other negatively impacted relationship dimensions (**S2 Table**). Approximately half of parents reported a negative impact on the time and activities with their partner, and the majority of them reported that these negative effects either disappeared immediately or faded with time (94.7% of mothers and 98% of fathers). Also, 68% of mothers and 57% of fathers reported that their child’s illness had a negative impact on their sexuality as a couple. Among these parents reporting negative effects on sexuality, 8.6% of mothers and 16.9% of fathers reported that the effects were permanent. Altogether, approximately three quarters of parents reported that the period in which their child was ill and treated for leukemia brought them closer together and strengthened their relationship (**Fig 1**). Additionally, the perceived impact of cancer on some relationship dimensions was associated with time since diagnosis, with the more time having passed, the greater the reported positive changes on the 1 to 7 Likert scale. Associations between time since diagnosis and perceived relationship changes were more pronounced among fathers than mothers (**S3 Table**).

### Objective 3: Dyadic models for long-term adjustment

Standardized regression coefficients from APIM models for all significant actor, partner, and gender effects are presented in **Table 3**. For both mothers and fathers, no relationship dimensions from the perceived impact of cancer were associated with their global distress. For mothers, greater current relationship satisfaction was associated with them perceiving several positive changes in their relationship with their partner following the illness (**Fig 2**), specifically on: intimacy, quality of partner support, sexuality, relationship satisfaction, and the overall impact of illness on the couple (actor effects). Partner effects for mothers were not statistically significant (*p* > .05). As for psychological distress, mothers reporting that the period of their child’s illness brought them closer and strengthened their relationship with their partner was associated with them self-reporting more current anxiety symptoms (actor effect). Moreover, the more positive changes they perceived that the child’s illness had on their intimacy with their partner, the fewer depression symptoms that mothers reported (actor effect).

**Table 3:** Actor, partner, and gender effects as identified by APIM models predicting relationship satisfaction and psychological distress from the perceived impact of cancer on the couple (n = 100).

	**Actor effect**			**Partner effect**		
**Predictors of relationship satisfaction**	**Mother**	**Father**	**Gender difference**	**Mother**	**Father**	**Gender difference**
**Impact of Cancer on Couple**						
Intimacy	*β* = .278*	*β* = .085	*p* = .237	*β* = .071	*β* = .197	*p* = .437
Quality of partner support	*β* = .337**	*β* = .068	*p* = .095	*β* = .067	*β* = .201*	*p* = .405
Sexuality	*β* = .293*	*β* = .102	*p* = .307	*β* = -.040	*β* = .199	*p* = .204
Conflict	*β* = .183	*β* = .161	*p* = .890	*β* = .192	*β* = .197*	*p* = .977
Relationship satisfaction	*β* = .559**	*β* = .201	*p* = .027	*β* = -.066	*β* = .288**	*p* = .028
Overall impact of illness	*β* = .453**	*β* = .188	*p* = .141	*β* = .086	*β* = .260*	*p* = .334
**Predictors of psychological distress**						
**Anxiety**						
Overall impact of illness	*β* = .272*	*β* = .220	*p* = .766	*β* = -.256	*β* = -.148	*p* = .541
**Depression**						
Intimacy	*β* = -.236*	*β* = .166	*p* = .009	*β* = .182	*β* = -.110	*p* = .056
Overall impact of illness	*β* = .233	*β* = .238*	*p* = .977	*β* = -.126	*β* = -.210	*p* = .643
**Somatization**						
Time & activities	*β* = .100	*β* = .0001	*p* = .499	*β* = -.027	*β* = .190*	*p* = .280
Overall impact of illness	*β* = -.031	*β* = .273*	*p* = .195	*β* = -.046	*β* = -.193	*p* = .434

*Note*. All possible associations were tested.

*p < .05

**p < .01

For fathers, their perceived relationship changes following cancer treatments were not significantly associated with their own current relationship satisfaction (i.e., no significant actor effects). Instead, fathers’ reported more relationship satisfaction when their partner’s reported positive changes on: quality of partner support, conflict, relationship satisfaction, and the overall impact of illness on the couple (partner effects). In terms of psychological distress, fathers reporting that the period of the child’s illness brought them closer to their partner and strengthened their relationship were associated with them reporting more current depression and somatization symptoms (actor effects). Moreover, when mothers reported that the illness had a primarily positive effect on the time and activities with their partner, fathers tended to report more current somatization symptoms (partner effect).

Finally, we found three significant gender differences (**Table 3**). First, when examining the association between current relationship satisfaction and the perceived changes in relationship satisfaction, we found a significant actor effect for mothers but this was not the case for fathers. Second, we found a significant partner effect for predicting fathers’ current relationship satisfaction from their partner’s perceived changes in relationship satisfaction. The opposite partner effect was not significant. Finally, the actor effect for mothers that related their current depression symptoms and their perceived impact of cancer on intimacy was significant, but this same association was not significant in fathers.

## Discussion

In an innovative follow-up study of a hundred and three couples of parents of long-term childhood ALL survivors, we found that only a small subset of parents reported clinical psychological and relationship distress five or more years (on average 15 years) following their child’s leukemia diagnosis. Prevalence of clinical levels of distress was lowest on psychological distress and highest on relationship distress. Generally, partners tended to agree on the nature of relationship changes experienced as a result of the cancer experience, but reported different levels of psychological symptoms. This finding may indicate that psychological distress is a unique experience for each partner, whereas relationship functioning is a communal and relatively similar experience for both partners.

### Description of long-term adjustment

Overall, we found that 2.9 to 21.4% of parents reported clinical levels of psychological or relationship distress on average 15 years following their child’s leukemia diagnosis. This range of clinical psychological distress found in our sample largely resembles the range found in a recent review of parents of childhood cancer survivors (compared to 8.8–30% of parents [4]). The proportion of parents that reported clinical levels of anxiety (5.8% of mothers and 6.8% of fathers) and depression symptoms (2.9% of mothers and 6.8% of fathers) in our study also largely resembles the proportions reported by another recent cross-sectional study on long-term acute lymphoblastic leukemia (ALL) survivors and their parents (compared to 7.1% of parents with clinical anxiety and 3.1% of parents with clinical depression [5]). With respect to somatization symptoms, our results (13.6% of mothers and 9.7% of fathers) are also similar to those of a previous study that found that 14% of parents of childhood survivors of solid and brain tumours reported clinical levels of somatization symptoms [22].

In terms of clinical relationship distress, the proportion of parents meeting the clinical threshold in our survivorship study (21.4% of mothers and 20.4% of fathers) is fairly similar to the proportions of parents meeting this threshold at diagnosis (25.5% of mothers and 21.3% of fathers) as indicated by a previous longitudinal study [9]. However, in that same longitudinal study, 36.2% of mothers and 42.6% of fathers reported clinically significant relationship distress two years later [9], suggesting that relationship adjustment may fluctuate depending on the course of the illness and treatment. That is, parents might report greater relationship distress two years after diagnosis (compared to the time of diagnosis and survivorship period) because this coincides with the end of their child’s treatments and the re-entry period. This is a time when specialized resources offered to the family are reduced and parents have to readjust to their child reintegrating into their normal family routine [37]. These changes may create relationship strain. This is congruent with the results of a cross-sectional study which showed that parents reported feeling the most emotionally connected to their partner at the time of their child’s diagnosis and the least emotionally connected to their partner at the beginning and end of treatment [8].

Next, we found that fathers reported significantly more depression symptoms than mothers several years after their child’s diagnosis. There were no other significant gender differences on global distress, anxiety, and somatization symptoms. This contrasts with previous findings on parental distress during treatment which indicate that mothers report significantly greater psychological distress [10, 38]. It is possible that mothers’ distress during treatment subsides with time so that their heightened distress is no longer apparent years after treatment, as was the case in our study. This proposition on the temporal nature of distress is coherent with findings from a longitudinal study on parents’ emotional functioning, which suggest that mothers’ distress levels largely resemble fathers’ distress levels once treatment has ended [39]. A cross-sectional study of mothers and fathers of children with cancer at 4 weeks to 14 years post-diagnosis also found that the time elapsed since diagnosis explained 2.2 to 13.9% of the variability in parents’ distress, with longer periods since diagnosis being associated with lower levels of distress [40].

### Description of perceived impact of cancer on couple

We found that couples differed with respect to their relationship adjustment post diagnosis, with some parents reporting primarily negative changes following the illness and others reporting positive changes. This is also coherent with findings from previous studies and reviews [6, 12, 41, 42]. Generally, we found that mothers and fathers had similar perceptions of relationship changes following cancer. Most parents tended to perceive that the illness period strengthened their relationship, suggesting that globally, the illness did not undermine their relationship as a couple, despite their perception that some specific areas of their relationship were negatively impacted by the illness. Parents also tended to report positive changes on their relationship satisfaction and the quality of support provided by their partner, as was found in previous reports [6–8, 12, 42]. The cancer experience itself might have brought them closer to jointly cope with the crisis as a team [42–44].

Nevertheless, the majority of mothers and fathers in our study reported negative changes on aspects of their sexuality, intimacy, and time and activities with their partner. As the child’s illness takes precedence over the parents’ relationship as a couple, their sexuality often gets pushed aside [6–8, 12]. Previous studies have not specifically examined changes in intimacy and the time spent with their partner and their activities done together. The negative effects that were reported on these two dimensions in our study could be explained by the fact that the child’s illness often requires parents to rearrange family responsibilities, with one parent being the primary caregiver for the ill child at the hospital and the other being responsible for the finances and rest of the family [42, 45]. This division of labour could lead them to feel more distant and less emotionally connected to each other. As for the negative impact on parents’ time and activities done with their partner, this could simply be explained by the fact that parents have less time for leisure activities and again illness-related responsibilities take precedence over spending alone time with one’s partner. Nonetheless, given that most parents reported that negative effects disappeared with time, this suggests that the experience of having one’s child diagnosed and treated for leukemia was not permanently detrimental for the couple and instead was more of a transient challenge that could be overcome with time.

Additionally, we found that the more time that had passed since the child’s diagnosis, the greater the likelihood that parents perceived their relationship changes as being more positive in nature, especially for fathers. This is conceptually coherent with the psychological adaptation process [46, 47], precisely since survivors were all in remission at the time of this study. It is important to note that the current relationship functioning of parents of ALL survivors in our sample may have biased their recollection of the dynamics of their relationship with their partner during their child’s treatment [48–50]. As most parents reported high current relationship satisfaction in our study, this may have tainted their recollection of past events, especially since for some parents the end of their child’s cancer treatments were up to 25 years ago.

### Impact of cancer on the relationship and long-term adjustment

The current study is the first to examine dyadic associations between mothers and fathers’ psychological and relationship adjustment in the survivorship period using predictors of perceived changes in relationship dynamics within couples as a result of the cancer experience. We found that mothers’ adjustment was exclusively self-related (actor effects), whereas fathers’ adjustment was mostly partner-related (partner effects) and to a lesser extent self-related. These results are both coherent with and extend results from previous studies in this field. Similar trends in actor and partner effects for mothers and fathers were reported in a recent dyadic, longitudinal study of parents of children with cancer in the first two years following their diagnosis [9]. That is, mothers’ marital adjustment 2 years later was explained by their own perceived family functioning at diagnosis (actor effects), whereas fathers’ marital adjustment was explained by their own mood and family functioning (actor effects), as well as by their partner’s family functioning at diagnosis (partner effects) [9].

#### Predicting relationship satisfaction

Parents of children with cancer are often well adjusted in their relationship with their partner [7], and this finding on general relationship functioning (e.g., relationship satisfaction) is consistent with what was found in our study. In the present study, we found that mothers’ current relationship satisfaction was associated with their own perceptions of positive relationship changes (intimacy, partner support, sexuality, satisfaction, and overall impact of the illness), whereas fathers’ current relationship satisfaction was associated with their partner’s perceived positive relationship changes (partner support, conflict, satisfaction, and overall impact of the illness). These findings seem to suggest that long-term relationship adjustment is an independent experience for mothers, but an interdependent experience for fathers. In this way, fathers’ relationship satisfaction might be at least partially dependent on how their partner views their relationship and the subsequent changes in their relationship dynamics following their child’s illness.

It is possible that this differential pattern in mothers and fathers could be due to their fundamental differences in support-seeking behaviours. Several studies have suggested that while mothers of children with cancer receive their social support from various sources (including their partner, friends, family, and the health care team), fathers primarily seek support from their partner [41, 51]. In fact, a cross-sectional study of parents of children with cancer (*n* = 35 couples) found that while mothers’ reliance on social support lowered their distress, fathers felt less distressed when their relationship with their partner was strong [41]. This tendency for fathers to largely depend on their partner for support, could in turn explain why mothers’ experiences during the child’s illness are consistently associated with fathers’ relationship adjustment even several years after their child’s diagnosis. Also, given that mothers are often the primary caregivers for the ill child and more frequently accompany the child at the hospital, it is possible that fathers use mothers’ experiences as a bridge for understanding their child’s cancer experience and its impact on the family.

#### Predicting psychological distress

Our results suggest that the changes that occurred in couples’ relationships during their child’s leukemia treatments are significantly related to both partners’ psychological distress during the survivorship period. Specifically, we found that mothers’ perception that the illness had a positive effect on their intimacy and that it strengthened their relationship with their partner was associated with them reporting less depression and more anxiety symptoms at the time of our follow-up study. Effects were also found for fathers, whereby their perception that the illness period brought them closer and strengthened their relationship was associated with them reporting greater current depression and somatization symptoms. We also found that certain relationship changes perceived by mothers (improved time and activities together) were associated with fathers reporting greater current somatization symptoms. At first glance these findings seem counterintuitive, as we would expect that recalling positive relationship changes during the child’s treatment would be associated with less distress later. Yet, given that this study was a cohort recall aimed at examining long-term adverse effects in ALL survivors, the very nature of this study could explain the present phenomenon. Since parents were asked to self-report psychological distress in the last week including on the day of their child’s follow up medical testing, parents’ heightened anxiety, depression, and somatization symptoms are understandable and thus may not be a representative depiction of their standard level of distress (e.g., trait anxiety). Instead it might reflect state-dependent distress, which is mostly related to their illness-related concerns regarding late effects and limitations that could be found in their child’s upcoming medical follow-up appointment. Furthermore, it is possible that self-reflecting on their child’s illness in the days leading up to the follow-up study, even when related to positive relationship effects, could have served as an inadvertent mood prime and bias for their subsequent reports of their psychological status.

### Limitations

Although the current study follows a relatively large cohort of parents whose children were treated for ALL for a long period of time, its cross-sectional nature prevents us from interpreting associations as causal links. For example, causality between perceptions of the impact of cancer on couples’ relationship and parents’ adjustment can only be hypothesized, as it is possible that retrospective evaluations of the impact would be influenced by their current psychological status. Furthermore, we should be mindful that our sample only reflects the experiences of relatively stable couples that were together both during their child’s illness and at the time of follow-up. This restrictive definition of couples excludes all mothers and fathers who have separated from the other parent and precludes including couples from reconstituted families (i.e., step-parents). Next, the homogeneity of diagnosis in this sample facilitates comparisons of the long-term effects and adjustment of survivors and their parents. However, since rates of survival for childhood ALL are relatively high, the reports of parents in this sample might not reflect the experiences of parents of survivors with more sombre survival expectations or morbidities, such as parents of children treated for brain tumour or those who had relapsed. Finally, given the final participation rate we cannot rule out the effect of a possible selection bias. Although we tried to include all parents of children treated with standard DFCI protocols from 1987 to 2005, those who did not respond or send back their questionnaires may be those that are less well-adjusted, as evidenced with greater psychological distress or more substantial relationship challenges.

### Conclusion

In a cross-sectional study of the retrospective adjustment experiences of 103 couples of parents of childhood ALL survivors, we found that parents were generally well-adjusted, with only a small subset (2.9–21.4%) reporting clinical distress at follow-up. Using an interdependence model, this study was the first to examine the dyadic adjustment and relationship change experiences of parents of childhood ALL survivors. In doing so, we found that mothers’ adjustment (relationship satisfaction and psychological distress) was solely associated with her own perceptions of changes in relationship dynamics, while fathers’ adjustment was associated with both their own perceptions and those of their partner. It is thus possible that by strengthening dimensions of relationship functioning among mothers during the time of the illness we would actually be fostering better long-term adjustment for both parents. This observation could be translated into new integrated and family-based approaches for addressing individual and interpersonal distress among parents. For instance, providing a couples-based support program which uses what other researchers have referred to as ‘relationship talk’ (i.e., partners discussing their relationship and dimensions within that relationship, [52]) or ‘social sharing’ (i.e., expressing their thoughts and feelings regarding cancer, [53]) during the child’s cancer treatments could help both partners address their interpersonal difficulties early in the illness trajectory. This would also allow mothers to openly address their perceptions of negative relationship changes with their partner. This in turn could help to foster stronger general relationship functioning for both partners and promote long-term relationship satisfaction and psychological well-being in this vulnerable population.

## Supporting information

S1 Fig Flowchart of study participants. *Note*. Depression variables were severely skewed, and parents in three couples had extreme depression scores (*z* score > 3.5). Data from these three couples were retained in all analyses for Objectives 1 and 2, but due to normality concerns they were excluded from the dyadic analyses in Objective 3.
(TIF)

S1 File Impact du cancer sur le couple. (PDF)

S2 File Impact of cancer on the couple. (PDF)

S1 Table Proportion of mothers and fathers scoring within the clinical range (i.e., positive caseness) on adjustment variables (*n* = 103). (PDF)

S2 Table Proportion of parents that reported negative change, no change and positive change in relationship dimensions on the Impact of cancer on the couple (n = 103). *Note*. Relationship dimensions are represented on a 1–7 scale. Classifications of scores are as follows: Scores 1–3 = negative effect, 4 = no effect, and 5–7 = positive effect. Bolded text indicates a significant gender difference.
(PDF)

S3 Table Correlations between perceived impact of cancer and adjustment variables in mothers and fathers of children treated for acute lymphoblastic leukemia (*n* = 103). *Note*. Mothers’ bivariate correlations are above the diagonal and fathers’ bivariate correlations are below the diagonal. Interrelationships between the partners’ variables are displayed on the diagonal. ** p < .01, * p < .05.
(PDF)

S1 Database PETALE database. (SAV)
