# Supplementary figures and images for: Cancer-related effects on relationships, long-term psychological status and relationship satisfaction in couples whose child was treated for leukemia: A PETALE study
Source: PLoS One. 2018 Sep 7;13(9):e0203435. doi: 10.1371/journal.pone.0203435 (PMC6128557; doi:10.1371/journal.pone.0203435)

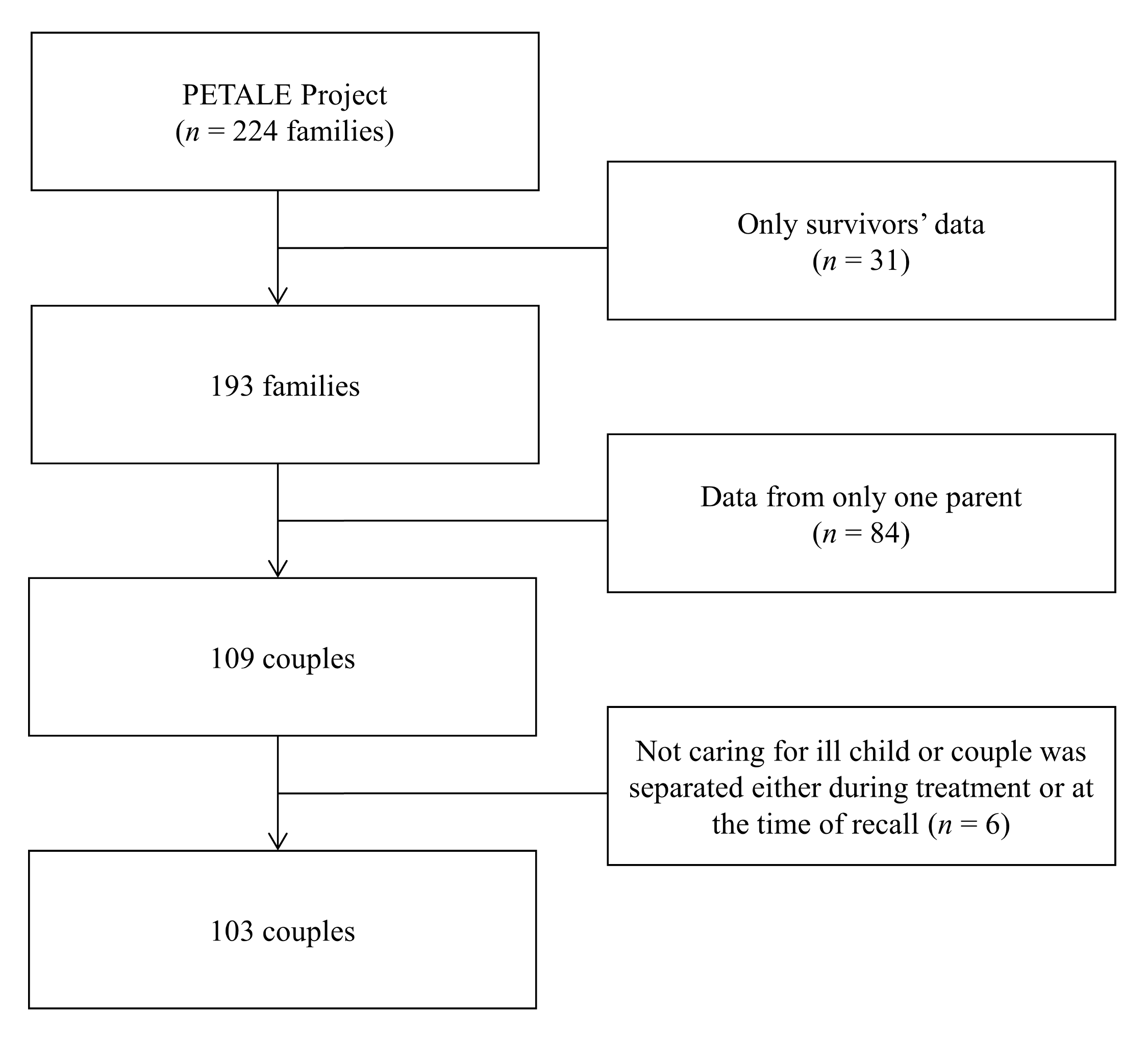

Supplement: S1 Fig — Note. Depression variables were severely skewed, and parents in three couples had extreme depression scores (z score > 3.5). Data from these three couples were retained in all analyses for Objectives 1 and 2, but due to normality concerns they were excluded from the dyadic analyses in Objective 3. (TIF) [file pone.0203435.s001.tif]
