# Supplementary material for: Cancer-related effects on relationships, long-term psychological status and relationship satisfaction in couples whose child was treated for leukemia: A PETALE study
Source: PLoS One. 2018 Sep 7;13(9):e0203435. doi: 10.1371/journal.pone.0203435 (PMC6128557; doi:10.1371/journal.pone.0203435)
Supplement: S1 File — (PDF) [file pone.0203435.s002.pdf]

IMPACT DU CANCER SUR LE COUPLE
(Peloquin & Sultan, 2013)

1. Quel âge aviez-vous au début de votre relation de couple avec le père / la mère de votre enfant survivant de cancer? _________________

2. Depuis combien de temps êtes-vous en couple (ou pendant combien de temps avez-vous été en couple) avec le père / la mère de votre enfant survivant de cancer? _________________

3. Quel est votre statut conjugal actuel?
    1. ___ En couple avec le père / la mère de mon enfant survivant de cancer (allez directement à la question 5)
    2. ___ En couple avec un(e) autre conjoint(e) (répondez à la question 4)
    3. ___ Seul(e) (séparé / divorcé) (répondez à la question 4)
    4. ___ Veuf / veuve (allez directement à la question 5)

4. Si vous êtes séparé(e) / divorcé(e) du père / de la mère de votre enfant survivant de cancer, à quel point la maladie de votre enfant a-t-elle contribué à cette séparation selon vous ?
    1. ___ La maladie (et le contexte entourant la maladie) n'est *pas du tout liée* à notre séparation.
    2. ___ La maladie (et le contexte entourant la maladie) a *légèrement* contribué à notre séparation.
    3. ___ La maladie (et le contexte entourant la maladie) a *modérément* contribué à notre séparation.
    4. ___ La maladie (et le contexte entourant la maladie) a *beaucoup* contribué à notre séparation.
    5. ___ La maladie (et le contexte entourant la maladie) est *entièrement responsable* de notre séparation.

5. Étiez-vous en couple avec le père / la mère de votre enfant survivant de cancer au moment où votre enfant était malade et suivi pour des traitements à Sainte-Justine?
    1. ___ Oui, nous étions en couple (répondez à la question 6)
    0. ___ Non, nous étions séparés (ne répondez pas aux questions 6 et 7)

Les questions suivantes traitent de la période pendant laquelle votre enfant était malade et suivi pour des traitements. SVP répondre à ces questions en repensant à votre relation de couple pendant cette période.

6. Lorsque les couples vivent des situations de stress important, incluant la maladie d'un enfant, il est possible que ces situations influencent leur relation de couple de diverses façons. À l'aide des échelles de réponse suivantes, veuillez évaluer à quel point la maladie de votre enfant a eu un effet sur les dimensions conjugales suivantes :

6.A.1 L'intimité entre vous et votre conjoint(e) :

1	*2*	*3*	*4*	*5*	*6*	*7*
Effet très négatif	*Effet modérément négatif*	*Effet légèrement négatif*	*Aucun effet*	*Effet légèrement positif*	*Effet modérément positif*	*Effet très positif*

6.A.2. Si vous avez répondu 1, 2 ou 3, ces effets négatifs ont-ils perduré lorsque les traitements de votre enfant ont été terminés ?

1. ___ Les effets négatifs ont disparu immédiatement
2. ___ Les effets négatifs ont perduré, mais se sont estompées avec le temps
3. ___ Les effets négatifs ont été permanents

6.B.1. La qualité du soutien entre vous et votre conjoint(e) :

1	*2*	*3*	*4*	*5*	*6*	*7*
Effet très négatif	*Effet modérément négatif*	*Effet légèrement négatif*	*Aucun effet*	*Effet légèrement positif*	*Effet modérément positif*	*Effet très positif*

6.B.2. Si vous avez répondu 1, 2 ou 3, ces effets négatifs ont-ils perduré lorsque les traitements de votre enfant ont été terminés ?

1. ___ Les effets négatifs ont disparu immédiatement
2. ___ Les effets négatifs ont perduré, mais se sont estompées avec le temps
3. ___ Les effets négatifs ont été permanents

6.C.1. La sexualité entre vous et votre conjoint(e) :

1	*2*	*3*	*4*	*5*	*6*	*7*
Effet très négatif	*Effet modérément négatif*	*Effet légèrement négatif*	*Aucun effet*	*Effet légèrement positif*	*Effet modérément positif*	*Effet très positif*

6.C.2. Si vous avez répondu 1, 2 ou 3, ces effets négatifs ont-ils perduré lorsque les traitements de votre enfant ont été terminés ?

1. ___ Les effets négatifs ont disparu immédiatement
2. ___ Les effets négatifs ont perduré, mais se sont estompées avec le temps
3. ___ Les effets négatifs ont été permanents

6.D.1. Les conflits entre vous et votre conjoint(e) :

1	*2*	*3*	*4*	*5*	*6*	*7*
Effet très négatif	*Effet modérément négatif*	*Effet légèrement négatif*	*Aucun effet*	*Effet légèrement positif*	*Effet modérément positif*	*Effet très positif*

6.D.2. Si vous avez répondu 1, 2 ou 3, ces effets négatifs ont-ils perduré lorsque les traitements de votre enfant ont été terminés ?

1. ___ Les effets négatifs ont disparu immédiatement
2. ___ Les effets négatifs ont perduré, mais se sont estompées avec le temps
3. ___ Les effets négatifs ont été permanents

6.E.1. Le temps passé et les activités réalisées avec votre conjoint(e) :

1	*2*	*3*	*4*	*5*	*6*	*7*
Effet très négatif	*Effet modérément négatif*	*Effet légèrement négatif*	*Aucun effet*	*Effet légèrement positif*	*Effet modérément positif*	*Effet très positif*

6.E.2. Si vous avez répondu 1, 2 ou 3, ces effets négatifs ont-ils perduré lorsque les traitements de votre enfant ont été terminés ?

1. ___ Les effets négatifs ont disparu immédiatement
2. ___ Les effets négatifs ont perduré, mais se sont estompées avec le temps
3. ___ Les effets négatifs ont été permanents

6.F.1. Votre satisfaction conjugale générale :

1	*2*	*3*	*4*	*5*	*6*	*7*
Effet très négatif	*Effet modérément négatif*	*Effet légèrement négatif*	*Aucun effet*	*Effet légèrement positif*	*Effet modérément positif*	*Effet très positif*

6.F.2. Si vous avez répondu 1, 2 ou 3, ces effets négatifs ont-ils perduré lorsque les traitements de votre enfant ont été terminés ?

1. ___ Les effets négatifs ont disparu immédiatement
2. ___ Les effets négatifs ont perduré, mais se sont estompées avec le temps
3. ___ Les effets négatifs ont été permanents

7. De façon générale, comment décririez-vous l'impact de la période pendant laquelle votre enfant était malade sur votre relation de couple ?

1	*2*	*3*	*4*	*5*	*6*	*7*
Cette période nous a éloigné / a été néfaste pour notre relation			*Cette période n'a eu aucun effet sur notre relation*			*Cette période nous a rapproché / a renforcé notre relation*
