# Supplementary material for: Cancer-related effects on relationships, long-term psychological status and relationship satisfaction in couples whose child was treated for leukemia: A PETALE study
Source: PLoS One. 2018 Sep 7;13(9):e0203435. doi: 10.1371/journal.pone.0203435 (PMC6128557; doi:10.1371/journal.pone.0203435)
Supplement: S2 File — (PDF) [file pone.0203435.s003.pdf]

IMPACT OF CANCER ON THE COUPLE

(Peloquin & Sultan, 2013)

1. How old were you at the beginning of your relationship with the father/mother of your child cancer survivor ? _________________

2. How long have you been a couple (or how for how long were you a couple) with the father/mother of your child cancer survivor ? _________________

3. What is your current marital status ?
   1. ___ In a relationship with the father/mother of my child cancer survivor (go directly to question 5)
   2. ___ In a relationship with another partner (answer question 4)
   3. ___ Single (separated/divorced) (answer question 4)
   4. ___ Widowed (go directly to question 5)

4. If you are separated/divorced from the father/mother of your child cancer survivor, in your opinion how much did your child's illness contributed to this separation ?
   1. ___ The illness (and the context surrounding the illness) is *not at all related to* our separation.
   2. ___ The illness (and the context surrounding the illness) *contributed slightly* to our separation.
   3. ___ The illness (and the context surrounding the illness) *moderately contributed* to our separation.
   4. ___ The illness (and the context surrounding the illness) *greatly contributed* to our separation.
   5. ___ The illness (and the context surrounding the illness) is *entirely responsible* for our separation

5. Were you in a relationship with the father / mother of your child cancer survivor at the time that your child was sick and followed for treatment at Sainte-Justine?
   1. ___ Yes, we were a couple (answer question 6)
   0. ___ No, we were separated (do not answer questions 6 and 7)

The following questions deal with the period in which your child was sick and followed for treatment. Please answer these questions, thinking back to your relationship during this period.

6. When couples experience severe stress, including a child's illness, these situations may influence their relationship in a variety of ways. Using the following response scales, please rate the extent to which your child's illness had an effect on the following relationship dimensions:

6.A.1 Intimacy between you and your partner:

1	2	3	4	5	6	7
Very negative effect	*Moderately negative effect*	*Slightly negative effect*	*No effect*	*Slightly positive effect*	*Moderately positive effect*	*Very positive effect*

6.A.2. If you answered 1, 2 or 3, did these negative effects persist when your child's treatments were completed?

1. ___ The negative effects disappeared immediately
2. ___ The negative effects have persisted, but have faded with time
3. ___ The negative effects were permanent

6.B.1. The quality of support between you and your partner:

1	2	3	4	5	6	7
Very negative effect	*Moderately negative effect*	*Slightly negative effect*	*No effect*	*Slightly positive effect*	*Moderately positive effect*	*Very positive effect*

6.B.2. If you answered 1, 2 or 3, did these negative effects persist when your child's treatments were completed?

1. ___ The negative effects disappeared immediately
2. ___ The negative effects have persisted, but have faded with time
3. ___ The negative effects were permanent

6.C.1. Sexuality between you and your partner:

1	2	3	4	5	6	7
Very negative effect	*Moderately negative effect*	*Slightly negative effect*	*No effect*	*Slightly positive effect*	*Moderately positive effect*	*Very positive effect*

6.C.2. If you answered 1, 2 or 3, did these negative effects persist when your child's treatments were completed?

1. ___ The negative effects disappeared immediately
2. ___ The negative effects have persisted, but have faded with time
3. ___ The negative effects were permanent

6.D.1. Conflicts between you and your partner:

1	2	3	4	5	6	7
Very negative effect	*Moderately negative effect*	*Slightly negative effect*	*No effect*	*Slightly positive effect*	*Moderately positive effect*	*Very positive effect*

6.D.2. If you answered 1, 2 or 3, did these negative effects persist when your child's treatments were completed?

1. ___ The negative effects disappeared immediately
2. ___ The negative effects have persisted, but have faded with time
3. ___ The negative effects were permanent

6.E.1. Time spent and activities done with your partner:

1	*2*	*3*	*4*	*5*	*6*	*7*
Very negative effect	*Moderately negative effect*	*Slightly negative effect*	*No effect*	*Slightly positive effect*	*Moderately positive effect*	*Very positive effect*

6.E.2. If you answered 1, 2 or 3, did these negative effects persist when your child's treatments were completed?

1. ___ The negative effects disappeared immediately
2. ___ The negative effects have persisted, but have faded with time
3. ___ The negative effects were permanent

6.F.1. Your overall relationship satisfaction:

1	*2*	*3*	*4*	*5*	*6*	*7*
Very negative effect	*Moderately negative effect*	*Slightly negative effect*	*No effect*	*Slightly positive effect*	*Moderately positive effect*	*Very positive effect*

6.F.2. If you answered 1, 2 or 3, did these negative effects persist when your child's treatments were completed?

1. ___ The negative effects disappeared immediately
2. ___ The negative effects have persisted, but have faded with time
3. ___ The negative effects were permanent

7. In general, how would you describe the impact of the period in which your child was sick on your couples' relationship?

1	*2*	*3*	*4*	*5*	*6*	*7*
This period has distanced us / has been detrimental to our relationship			*This period had no effect on our relationship*			*This period has brought us closer / strengthened our relationship*
