# Supplementary material for: Cancer-related effects on relationships, long-term psychological status and relationship satisfaction in couples whose child was treated for leukemia: A PETALE study
Source: PLoS One. 2018 Sep 7;13(9):e0203435. doi: 10.1371/journal.pone.0203435 (PMC6128557; doi:10.1371/journal.pone.0203435)
Supplement: S1 Table — (PDF) [file pone.0203435.s004.pdf]

S1 Table. Proportion of mothers and fathers scoring within the clinical range (i.e., positive caseness) on adjustment variables (n = 103).

	Couples				
	Mothers		Fathers		
**Individuals within clinical range**	***n***	**%**	***n***	**%**	**McNemar tests**
**Dyadic Adjustment Scale (DAS-4)**					
Relationship satisfaction	22	21.4%	21	20.4%	p = 1.000
**Brief Symptom Inventory (BSI-18)**					
Global Symptom Index	7	6.8%	8	7.8%	p = 1.000
Anxiety	6	5.8%	7	6.8%	p = 1.000
Depression	3	2.9%	7	6.8%	p = .344
Somatization	14	13.6%	10	9.7%	p = .503
