# Supplementary material for: Cancer-related effects on relationships, long-term psychological status and relationship satisfaction in couples whose child was treated for leukemia: A PETALE study
Source: PLoS One. 2018 Sep 7;13(9):e0203435. doi: 10.1371/journal.pone.0203435 (PMC6128557; doi:10.1371/journal.pone.0203435)
Supplement: S2 Table — Note. Relationship dimensions are represented on a 1–7 scale. Classifications of scores are as follows: Scores 1–3 = negative effect, 4 = no effect, and 5–7 = positive effect. Bolded text indicates a significant gender difference. (PDF) [file pone.0203435.s005.pdf]

S2 Table. Proportion of parents that reported negative change, no change and positive change in relationship dimensions on the Impact of Cancer on the Couple (*n* =103).

	Couples						
	Mothers			Fathers			
**Relationship Dimensions**	**Nature of changes**			**Nature of changes**			
Duration of the negative change	**Negative change (%)**	**No change (%)**	**Positive change (%)**	**Negative change (%)**	**No change (%)**	**Positive change (%)**	**Wilcoxon tests**
**Intimacy**	65.0	9.7	25.2	43.7	21.4	35.0	**z = - 2.861, p = .004**
Disappeared immediately (%)	31.3			33.3			
Faded with time (%)	62.7			64.4			
Permanent (%)	6.0			2.2			
**Quality of partner support**	7.8	10.7	81.6	3.9	13.6	82.5	z = - .932, p = .351
Disappeared immediately (%)	25.0			50.0			
Faded with time (%)	62.5			25.0			
Permanent (%)	12.5			25.0			
**Sexuality**	68.0	19.4	12.6	57.3	32.0	10.7	z = -1.110, p = .267
Disappeared immediately (%)	32.9			39.0			
Faded with time (%)	58.6			44.1			
Permanent (%)	8.6			16.9			
**Conflict**	32.0	45.6	22.3	26.2	52.4	21.4	z = - .642, p = .521
Disappeared immediately (%)	33.3			59.3			
Faded with time (%)	60.1			33.3			
Permanent (%)	6.1			7.4			
**Time & activities**	55.3	21.4	23.3	48.5	27.2	24.3	z = -.683, p = .495
Disappeared immediately (%)	39.3			54.0			
Faded with time (%)	55.4			44.0			
Permanent (%)	5.4			2.0			
**Relationship satisfaction**	30.1	17.5	52.4	22.3	25.2	52.4	z = - 1.091, p = .275
Disappeared immediately (%)	22.6			30.4			
Faded with time (%)	71.0			56.5			
Permanent (%)	6.5			13.0			
**Overall impact of illness on relationship**	19.4	7.8	72.8	12.6	13.6	73.8	z = -1.263, p = .207

Note. Relationship dimensions are represented on a 1 - 7 scale. Classifications of scores are as follows: Scores 1 - 3 = negative effect, 4 = no effect, and 5 - 7 = positive effect. Bolded text indicates a significant gender difference.
