# Supplementary material for: Cancer-related effects on relationships, long-term psychological status and relationship satisfaction in couples whose child was treated for leukemia: A PETALE study
Source: PLoS One. 2018 Sep 7;13(9):e0203435. doi: 10.1371/journal.pone.0203435 (PMC6128557; doi:10.1371/journal.pone.0203435)
Supplement: S3 Table — Note. Mothers’ bivariate correlations are above the diagonal and fathers’ bivariate correlations are below the diagonal. Interrelationships between the partners’ variables are displayed on the diagonal. ** p < .01, * p < .05. (PDF) [file pone.0203435.s006.pdf]

S3 Table. Correlations between perceived impact of cancer and adjustment variables in mothers and fathers of children treated for acute lymphoblastic leukemia (*n* = 103).

	**1**	**2**	**3**	**4**	**5**	**6**	**7**	**8**	**9**	**10**	**11**	**12**	**13**
**1.** Time elapsed since diagnosis		.147	.083	.172	-.023	.205*	.030	.046	-.131	.074	.055	.059	.116
**Impact of Illness**													
**2.** Intimacy	.216*	.327**	.423**	.657**	.404**	.570**	.562**	.477**	.283**	-.099	-.035	-.145	-.047
**3.** Quality of partner support	.121	.286**	.317**	237*	.476**	.275**	.525**	.553**	.369**	.077	.165	.086	.024
**4.** Sexuality	.257**	.602**	.083	.517**	.213*	.620**	.465**	.342**	.272**	-.059	-.047	-.136	.026
**5.** Conflict	.109	.432**	.285**	.412**	.366**	.244*	.435**	.534**	.267**	.141	.189	.152	.044
**6.** Time & activities	.315**	.412**	.133	.457**	.499**	.197*	.354**	.284**	.120	.055	.058	-.008	.106
**7.** Relationship satisfaction	.154	.469**	.476**	.488**	.450**	.437**	.472**	.616**	.565**	-.083	-.020	-.080	-.085
**8.** Overall impact of illness	.031	.393**	.500**	.283**	.368**	.319**	.634**	.543**	.511**	.105	.137	.132	.003
**Dyadic Adjustment Scale (DAS-4)**													
**9.** Relationship satisfaction	-.007	.149	.140	.206*	.252*	.196*	.333**	.331**	.661**	-.191	-.103	-.236*	-.164
**Brief Symptom Inventory (BSI-18)**													
**10.** Global Severity Index (GSI)	-.126	.071	.002	.049	-.049	-.012	.036	.145	-.208*	.142	.869**	.758**	.825**
**11.** Anxiety	-.184	.142	.034	.092	-.010	.016	.051	.154	-.154	.847**	.037	.593**	.599**
**12.** Depression	-.112	.139	.015	.089	-.072	-.029	.014	.134	-.181	.826**	.694**	.122	.475**
**13**. Somatization	-.047	.033	.020	.085	-.007	.031	.103	.171	-.120	.848**	.570**	.620**	.230*

Note. Mothers' bivariate correlations are above the diagonal and fathers' bivariate correlations are below the diagonal. Interrelationships between the partners' variables are displayed on the diagonal. ** $p < .01$, * $p < .05$
